# A Case Report of Hemophagocytic Syndrome

**DOI:** 10.7759/cureus.79150

**Published:** 2025-02-17

**Authors:** Tânia F Mendes, Ana Isabel Oliveira, Carolina Gomes, Nuno A Sousa, João Gonçalves Pereira

**Affiliations:** 1 Internal Medicine, Hospital Vila Franca de Xira, Vila Franca de Xira, PRT; 2 Intensive Care Unit, Hospital Vila Franca de Xira, Vila Franca de Xira, PRT

**Keywords:** cytomegalovirus (cmv), differential diagnosis, early diagnosis and treatment, hemophagocytic syndrome, systemic involvement

## Abstract

Hemophagocytic syndrome (HPS) represents a critical and often overlooked hyperinflammatory condition that can lead to rapid multi-organ failure and high mortality rates, particularly in adults. This article presents a compelling case study of a 45-year-old male with a complex clinical presentation, highlighting the diagnostic challenges posed by HPS, including its nonspecific symptoms and the necessity for a high index of suspicion. We underscore the paramount importance of early recognition, thorough differential diagnosis, and prompt initiation of treatment to improve patient outcomes. This case not only illustrates the intricacies of diagnosing HPS but also advocates for increased awareness among healthcare providers to mitigate the risks associated with this life-threatening syndrome.

## Introduction

Hemophagocytic syndrome (HPS) is a life-threatening condition characterized by a hyperinflammatory response, leading to systemic involvement and multi-organ dysfunction [1]. It is marked by an exaggerated immune response often triggered by infections, malignancies or autoimmune diseases. Although traditionally recognized in pediatric populations, the incidence of HPS in adults has gained increasing attention, particularly given its potential for rapid progression and high mortality rates - estimated to be as high as 88% if not diagnosed and treated promptly [2,3].

The pathophysiology of HPS involves a complex interplay of immune cells, particularly T cells and macrophages, which become dysregulated, leading to excessive cytokine release [4,5]. This results in significant inflammation and can manifest with symptoms such as fever, cytopenias, hepatosplenomegaly, and elevated levels of ferritin and other inflammatory markers. Due to its nonspecific presentation, HPS is frequently misdiagnosed or underdiagnosed, complicating timely intervention [2,3].

The challenge lies not only in recognizing the syndrome but also in identifying its underlying etiology. HPS can be classified as primary, often stemming from genetic predispositions, or secondary, which is more prevalent in adults and can arise from a variety of triggers [2,3,6]. Common secondary causes include viral infections (such as Epstein-Barr virus and cytomegalovirus), malignancies (especially lymphomas), and autoimmune conditions (notably, macrophage activation syndrome [MAS] associated with systemic lupus erythematosus) [2,3,6,7].

Early identification is crucial for effective management, as the therapeutic approach may vary significantly depending on the underlying cause [2,3,8,9]. While high ferritin levels serve as key diagnostic indicators [9], additional markers, such as soluble CD25, can provide further confirmation [8, 9]. This article aims to highlight the importance of early diagnosis and the intricacies involved in the differential diagnosis of HPS, using a clinical case to illustrate the diagnostic challenges encountered in practice.

## Case presentation

A 45-year-old man presented to the emergency department with a 5-day history of fever (maximum axillar temperature of 39ºC), night sweats and fatigue. His clinical background was remarkable for systemic lupus erythematosus (SLE) with secondary antiphospholipid syndrome, diabetes *mellitus* secondary to the corticosteroids and Hashimoto's thyroiditis with hypothyroidism. He was currently receiving acenocoumarol, levothyroxine, acetylsalicylic acid and metformin. After the diagnosis of SLE, he received corticosteroids (suspended 10 years ago) and hydroxychloroquine, which was suspended at the age of 42, due to eye toxicity. He remained clinically stable with no direct SLE therapy.

On admission, he presented inguinal adenopathies, the biggest with 5cm, adherent to deep planes, hard consistency and painful to palpation; hepatomegaly, palpable 1cm below the costal border. Blood tests were remarkable for thrombocytopenia, elevated C-reactive protein (CRP), discrete elevated liver enzymes (cytocholestatic pattern), and elevated lactic dehydrogenase (LDH) (Table 1).

**Table 1:** Most relevant blood test results over time Highlighted are the altered results, regarding the reference values. Treatment with dexamethasone was initiated 17 days after admission. The patient was discharged from the hospital 22 days after admission. ALP, Alkaline phosphatase; ALT, Alanine transaminase; AST, Aspartate aminotransferase; CRP, C-reactive protein; GGT, Gamma-glutamyl transferase; Hb, Hemoglobin; LDH, Lactate dehydrogenase; SV, Sedimentation velocity; TB, Total bilirubin

Laboratorial parameters	Days after admission								Time after discharge			Reference range
	0	1	2	5	8	10	15	22	7 days	21 days	5 months	
Hb (g/dL)	13.3	13.4	13.3	13.5	13.6	11.8	9.8	11.4	10.9	12.3	13.8	13.0 – 17.0
Leucocytes (10^3^/µL)	4.4	5.1	4.5	8.4	14.0	24.2	11.6	11.7	13.7	10.3	6.7	4.0 – 10.0
Lymphocytes (10^3^/µL)	1.1	1.2	1.2	4.2	6.7	9.9	8.1	3.0	2.3	4.8	3.6	0.8 – 4.0
Platelets (10^3^/µL)	135	138	149	238	295	307	394	433	455	265	211	150 – 400
AST (UI/L)	54	106	237	495	-	-	-	43	25	21	17	15 – 37
ALT (UI/L)	70	115	227	492	-	-	-	88	66	66	39	16 – 63
GGT (UI/L)	121	180	277	723	-	-	-	462	281	93	48	15 – 85
ALP (UI/L)	185	295	460	1547	-	-	-	642	307	97	87	50 – 136
TB (mg/dL)	0.65	0.75	0.88	1.79	-	-	-	0.83	0.48	-	0.8	<1
LDH (UI/L)	505	601	-	-	-	637	-	206	152	190	138	85 – 227
CRP (mg/dL)	17.9	22.0	20.9	15.8	11.8	10.0	3.8	0.7	-	-	-	<1
SV (mm)	-	-	32	-	-	12	-	-	-	-	8	0 – 15
Triglycerides (mg/dL)	-	174	-	-	-	534	-	259	-	-	-	<150
Fibrinogen (mg/dL)	-	-	-	-	-	125	-	268	173	273	221	210 – 400
Ferritin (ng/mL)	-	-	-	-	-	2978	-	-	-	106	64	30 – 340

**Figure 1:**
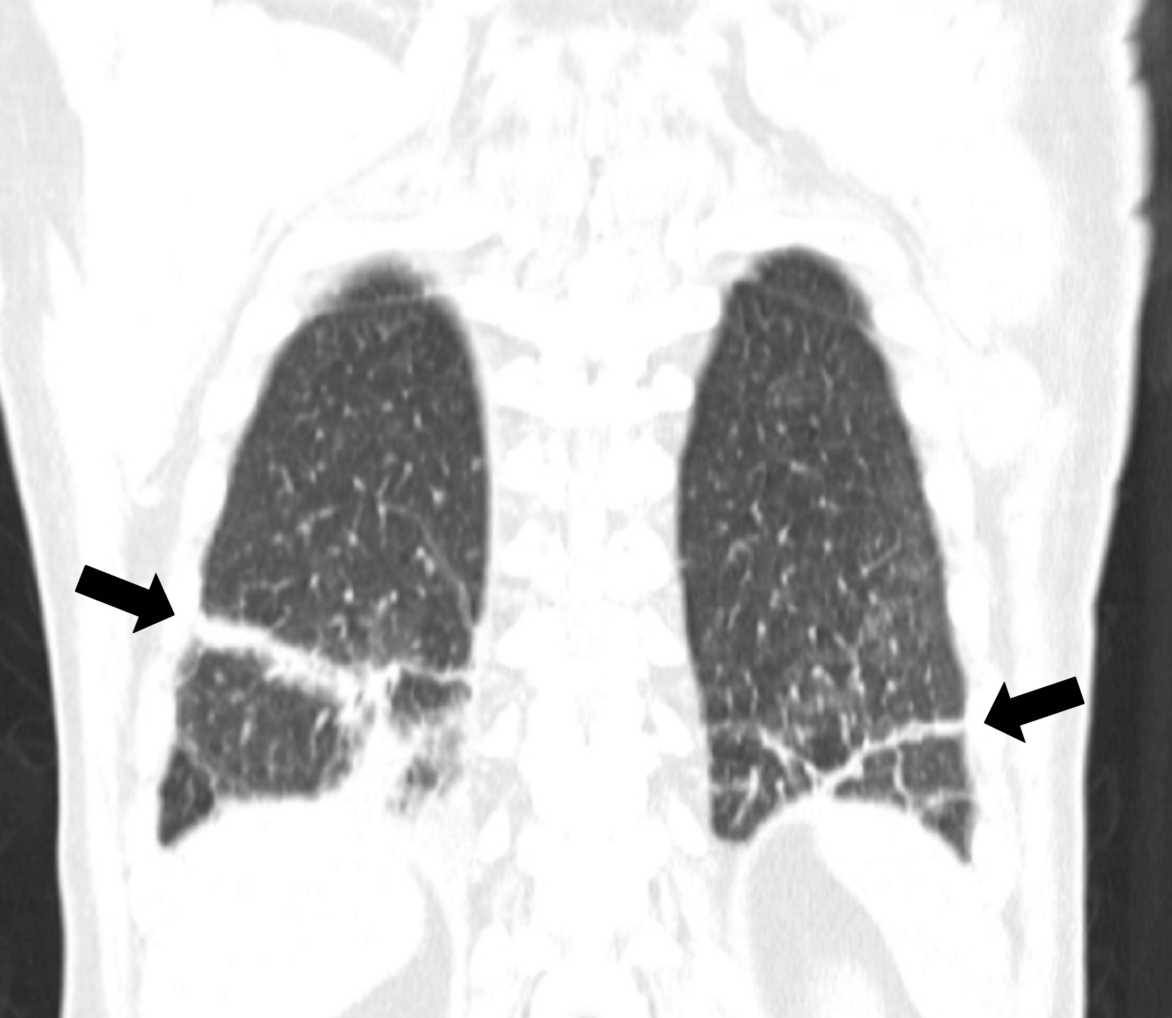
Chest CT at admission Chest CT at hospital admission showing interstitial inflammatory infiltrate in lung bases.

A computed tomography (CT) stood out interstitial inflammatory infiltrate in lung bases (Figure 1), homogenous hepatosplenomegaly and multiple scattered adenopathies (mediastinal, hilar, axillary, retroperitoneal, iliac and inguinal chains, the last ones larger, measuring up to 4.5cm in diameter).

A positive serology for cytomegalovirus was found with a high serum viral load (more than 230000 copies/mL); serologies for human immunodeficiency virus, Epstein-Barr, hepatitis B and C, and Venereal Disease Research Laboratory were all negative. An excisional biopsy of the larger adenopathy was performed and revealed reactive lymphadenitis; positive antinuclear antibodies but no complement consumption was found (C3 123mg/dL and C4 44mg/dL) alongside low elevated sedimentation velocity (Table 1).

**Figure 2:**
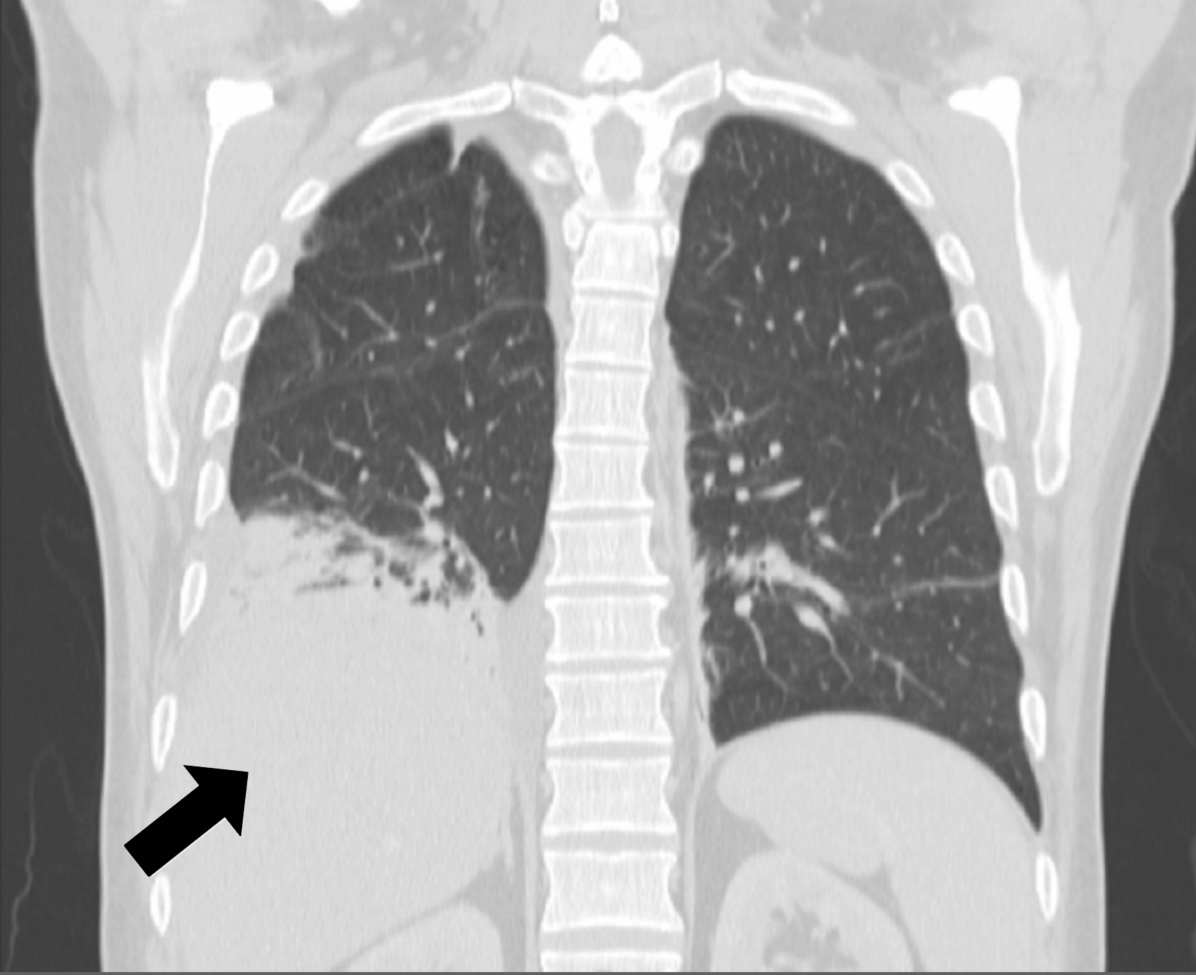
Chest CT on the 8th day of hospitalization, coronal view. Chest CT on the 8th day of hospitalization, corresponding to the patient's clinical deterioration, showing new consolidation in the right lung.

**Figure 3:**
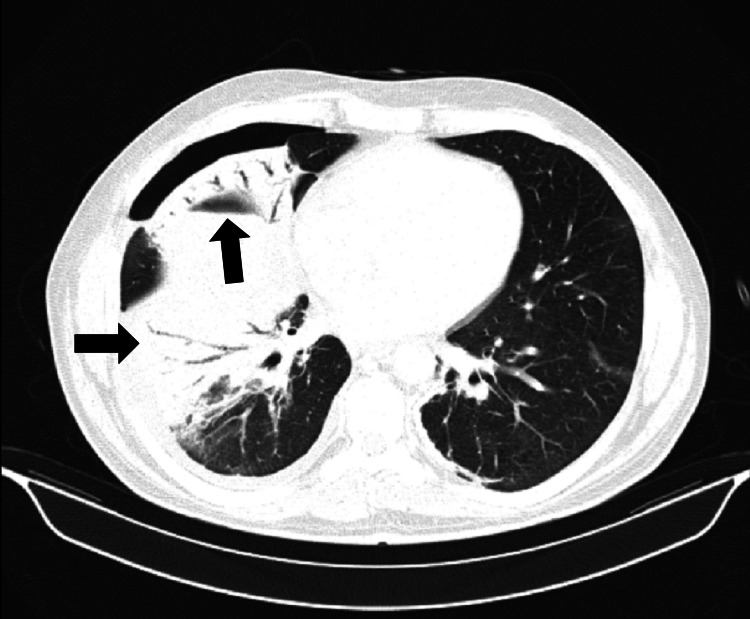
Chest CT on the 8th day of hospitalization, axial view. Chest CT on the 8th day of hospitalization, corresponding to the patient's clinical deterioration, showing new consolidation in the right lung with an air bronchogram with loss of volume at the middle lobe.

**Figure 4:**
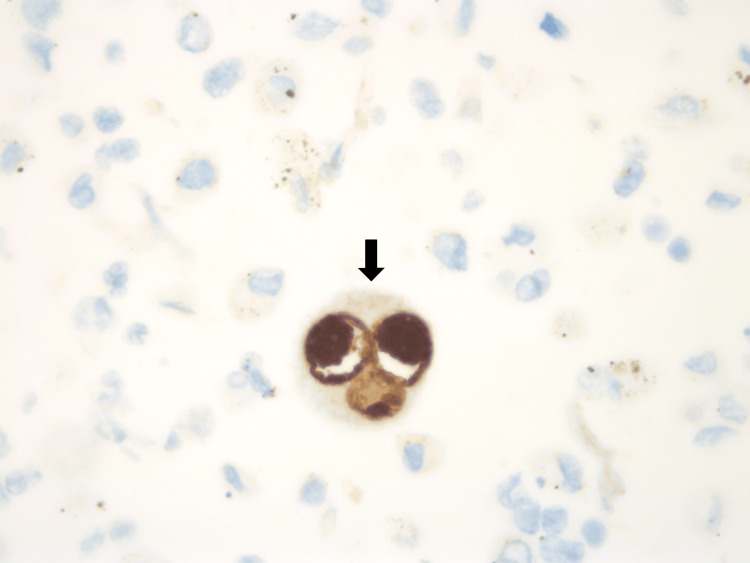
Cytomegalovirus (CMV) immunohistochemical stain on bronchoalveolar lavage. The infected cells with CMV inclusions are highlighted in brown color.

**Figure 5:**
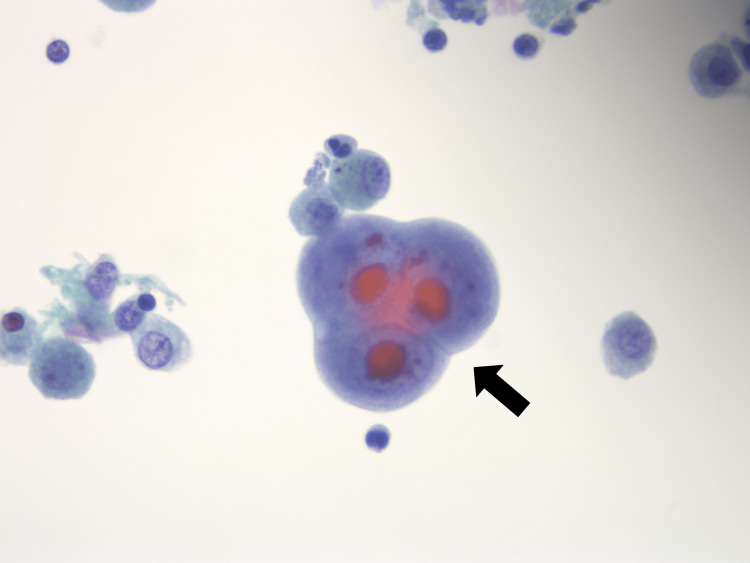
Cytomegalovirus (CMV) immunohistochemical stain, on bronchoalveolar lavage. The infected cells with CMV intranuclear inclusions are highlighted in red color.

**Figure 6:**
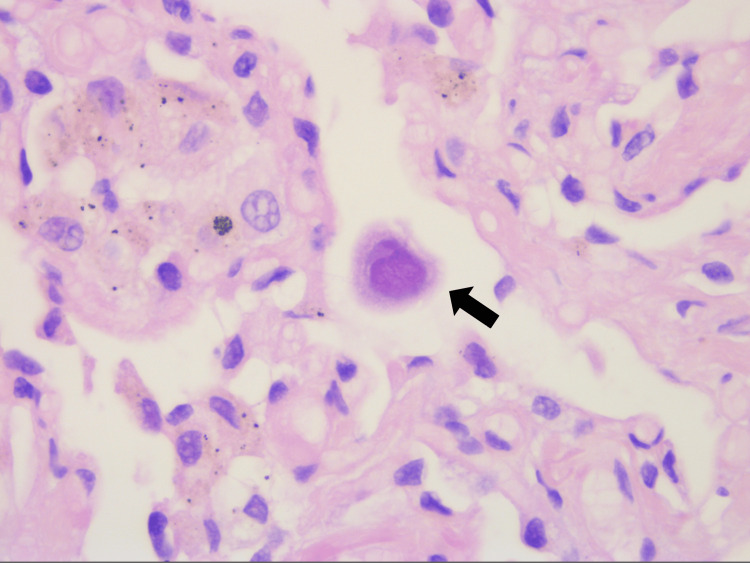
Cytomegalovirus immunohistochemical stain, on lung biopsies. Showing a large cell with a voluminous intranuclear inclusion.

The fever resolved spontaneously after four days of admission. However, on the eighth day of hospitalization, fever recurred (38-39ºC), now accompanied by dyspnea, hypoxemia, gingival edema and bleeding. Decreased right lung sounds were described. Blood tests revealed progressive leukocytosis with lymphocytosis. At this stage, there was a decreasing CRP concentration, rising LDH levels, worsening liver enzymes (same cytocholestatic pattern), hypertriglyceridemia and reduced fibrinogen (Table 1). A test for ferritinemia showed a very high concentration (2978ng/mL). A chest X-ray unveiled a right pleural effusion that was sent for cytological and microbiological study. The chest CT scan (Figures 2, 3) demonstrated de novo condensation on the right lung with air bronchogram, with loss of volume at the middle lobe and in both lung bases, maintaining the same nonspecific nodes. Bronchofibroscopy with bronchoalveolar lavage and lung biopsies were performed and confirmed inclusions of cytomegalovirus (Figures 4-6). Microbiological tests were negative (including virus PCR).

Towards the rapid evolution and systemic involvement, an exuberant, anomalous inflammatory reaction, more precisely HPS was considered. The patient had four HLH-2004 diagnostic criteria, one shorter than necessary for the diagnosis. There were also other suggestive factors (although not diagnostic) such as increased hepatic transaminases, increased LDH, hepatomegaly and hemorrhagic dyscrasia. Owing to the patient's rapid clinical deterioration, empirical treatment with dexamethasone was started, according to recommendations [3].

A myelogram and bone biopsy were performed, and a revision of the inguinal ganglion was requested. Both proved to be negative for hemophagocytic activity but revealed a soluble CD25 receptor abnormally high (5750U/mL; reference value: 229-999U/mL).

A diagnostic of HPS was confirmed which was deemed secondary to CMV infection (pulmonary, pleural, hepatic and lymph node involvement).

After starting corticosteroids, clinical improvement was rapidly noted, including regression of fever, adenopathies, gingival bleeding and hypoxemia. There was also a good analytical response (Table 1).

The patient was discharged from the hospital five days after the start of dexamethasone, asymptomatic. During the follow-up, normalization of all analytical parameters (Table 1), as well as regression of adenopathies and pulmonary involvement on CT scan were recorded.

## Discussion

This case exemplifies the complex challenges of diagnosing HPS in a clinical setting, where nonspecific symptoms can easily be attributed to more common conditions, such as infections or exacerbations of autoimmune diseases. The patient initially presented with fever, adenopathies, and fatigue, prompting a broad differential diagnosis that included lymphoproliferative disorders and infections. However, the emergence of new symptoms - specifically respiratory distress and gingival bleeding - raised significant clinical concerns. This rapid deterioration underscored the importance of ongoing clinical vigilance and collaboration among specialists, which is critical for managing complex cases like HPS.

The HLH-2004 diagnostic criteria are represented in Table 2 [10]. Although these criteria are critical in the diagnostic process, the most important factor remains a thorough clinical evaluation of the patient, as there are other factors - such as liver dysfunction, elevated LDH and the severity and progression of symptoms, among others - that can contribute to the diagnosis [8]. In complex cases like this, careful monitoring and a holistic, multidisciplinary approach are essential to accurately identify and manage HPS.

**Table 2:** HLH-2004 diagnostic criteria Adapted from *Hemophagocytic Syndrome-An Approach to the Management*, by Salunke B, Savarkar S, Patil VP [10]. This article is distributed under the terms of the Creative Commons Attribution 4.0 International License (https://creativecommons.org/licenses/by-nc/4.0/). BM, bone marrow; Hb, Hemoglobin; NK, natural killer

HLH-2004 Diagnostic Criteria
Molecular diagnosis - Biallelic pathogenic variants in any one of PRF1, UNC13D, STX11, or STXBP2
OR
5 out of 8 from the following:
(1) Prolonged fever (>7 days)
(2) Splenomegaly
(3) Cytopenias (≥2 lineages of peripheral blood) - Hb<9g/dL; Platelets <100,000/µl; Neutrophils <1000/µl
(4) Triglycerides ≥265mg/dL and/or Fibrinogen <150mg/dL
(5) Evidence of hemophagocytes on BM/spleen/lymphatic nodes - Without evidence of malignancy
(6) Diminution/absence of NK-cells activity - With normal circulating NK cells number
(7) Serum ferritin ≥500ng/mL
(8) High plasma concentrations of soluble CD25 ≥2400U/mL

One of the important findings in this case was the high ferritin level (Table 1), which is strongly associated with HPS [11]. Elevated ferritin levels are a key diagnostic marker, often indicating severe inflammation, and are particularly important in the context of HPS, where hyperferritinemia is commonly observed [9,11,12]. This case further demonstrated the diagnostic utility of the elevated soluble CD25 receptor, which reinforced the suspicion of HPS despite the absence of hemophagocytosis in initial biopsies. This aspect of HPS is crucial - histopathological confirmation of hemophagocytosis is not always present in the early stages, and the absence of this feature does not exclude the diagnosis [12-14].

The importance of early diagnosis in HPS is further emphasized by the high mortality rate, which can reach up to 88% in untreated cases [2,3,13].

HPS often presents diagnostic challenges due to its overlap with other conditions such as sepsis and autoimmune exacerbations. This case highlights the need for clinicians to maintain a high index of suspicion in patients presenting with systemic inflammation [10,15].

The subsequent rise in inflammatory markers, particularly ferritin, as well as other analytic parameters (Table 1), guided the clinicians towards the correct diagnosis. Monitoring these markers throughout the patient's course allowed for an effective evaluation of disease progression and treatment response.

Several reviews [14,15] describe how the syndrome can progress rapidly, leading to multi-organ failure and death. Mattos et al. conducted a retrospective review of 45 cases of HPS, where patients received no specific treatment and were managed solely for their underlying conditions. The mortality rate was 35.5%, with a median survival of 23 months (95% CI: 22-60). Most fatalities occurred within the first two months [14].

Abdelhay et al. [16] analyzed data from the National Inpatient Sample to assess inpatient treatment practices for HPS from 2007 to 2019 and their association with clinical outcomes. Patients were categorized into two groups: early treatment (<6 days) and late treatment (≥6 days). Results showed that delayed treatment was associated with higher in-hospital mortality (OR 2.00), circulatory shock (OR 1.33), mechanical ventilation (OR 1.41), venous thromboembolism (OR 1.70), infectious complications (OR 2.24), acute kidney injury (OR 2.27), and the need for hemodialysis (OR 1.45). The study underscores the critical importance of early HPS treatment initiation to avoid adverse outcomes.

Early identification and prompt intervention, as seen in this case with the timely administration of corticosteroids (dexamethasone), are vital to mitigate the hyperinflammatory state that defines HPS. The rapid improvement following corticosteroid therapy aligns with findings from the literature, which emphasizes the importance of early therapeutic action to prevent irreversible organ damage [3,8,10,14].

Rosée et al. [3] outlined treatment strategies based on the HLH-2004 protocol, emphasizing that therapy should be individualized according to the patient’s clinical status and underlying etiology. For stable patients, the focus is on treating the underlying cause, while for unstable patients, the recommended approach includes dexamethasone with or without etoposide and/or intravenous immunoglobulin. HPS can result from various etiologies, including malignant (neoplastic or chemotherapy-induced), infection (e.g., viral, intracellular bacteria, HIV), or MAS. Specific treatments for these causes may involve corticosteroids, etoposide, rituximab, antibiotics, antiretroviral therapy, and immunosuppressants like cyclosporine A or anakinra. For refractory cases, rescue therapies such as alemtuzumab, ruxolitinib, and allogeneic transplantation are considered.

Early diagnosis and treatment of HPS not only improve immediate survival but also play a pivotal role in long-term recovery and quality of life [1,10,13]. The patient’s rapid clinical improvement and discharge following corticosteroid therapy underscore the potential for positive outcomes when HPS is recognized and treated early.

## Conclusions

This case underscores the paramount importance of early recognition and intervention in HPS. The high mortality rate, rapid progression and potential for severe complications make it essential for clinicians to remain vigilant in patients with systemic inflammatory symptoms. In this case, despite the absence of hemophagocytosis in initial biopsies, markedly elevated ferritin and soluble CD25 were critical in confirming the diagnosis, reinforcing the need to rely on multiple diagnostic parameters beyond histological findings. The favorable outcome in this case reinforces the critical nature of early diagnosis and treatment in managing HPS, ultimately improving both short- and long-term patient outcomes.
